# Supplementary material for: Secure image communication based on two-layer dynamic feedback encryption and DWT information hiding
Source: PLoS One. 2024 Aug 27;19(8):e0300228. doi: 10.1371/journal.pone.0300228 (PMC11349087; doi:10.1371/journal.pone.0300228)
Supplement: Attachment — Submitted filename: Response.docx [file pone.0300228.s001.docx]

**Response to Reviewer 1**

**[General Comment]** In this manuscript, the authors propose a secure image encryption scheme based on the 2D-SLMM chaotic system and a dynamic feedback mechanism. Overall, the manuscript is well-organized and well-written, and the work presented is both innovative and interesting. However, before accepting this manuscript for publication, the following minor issues need to be addressed:

**Response:** Thank you very much for the valuable time you have allocated to review this manuscript. We are honored to receive your suggestions and are very willing to make improvements to this article!

**[Comment 1]** Considering the length of the article, the authors are advised to carefully proofread the manuscript to remove any possible minor errors, such as typos.

**Response:** Thank you for your suggestion. We have carefully reviewed the entire article and made necessary proofreading and modifications to eliminate any potential spelling and other minor errors.

**[Comment 2]** The authors are advised to adjust the content of their abstract to more attractively present the motivations, methods, results, and conclusions of their work.

**Response:** Thank you for the suggestion. We have adjusted the content of the abstract to more attractively present the motivations, methods, results, and conclusions of our work.

**[Comment 3]** The manuscript comprises a few lengthy sentences, and the authors should contemplate modifying them to enhance readability.

**Response**: Thank you for your valuable suggestion. We have carefully reviewed the issue you raised regarding lengthy sentences in the manuscript and have made corresponding modifications to enhance overall readability.

 **[Comment 4]** It is recommended that Table 1 be appropriately adjusted to better present relevant content.

**Response:** Thank you for your suggestion. Table 1 has been appropriately adjusted to better present the relevant content. The modified table now provides information more clearly, facilitating better understanding for readers.

**[Comment 5]** Similarly, it is recommended that the figures in the manuscript be reviewed and adjusted to better present relevant content.

**Response:** Thank you for your suggestion. We have thoroughly reviewed and adjusted the figures in the manuscript to better present the relevant content. Following these corrections, the figures now convey information more clearly, aiding in a better understanding of the paper's content.

**[Comment 6]** The authors are advised to provide a more detailed explanation of the motivation, reasons, and purpose behind the utilization of the MD5 hash function.

**Response:** The MD5 function, as a type of hashing algorithm, plays a crucial role in data security. It can transform data of any length into a fixed-length hash value and is widely utilized in data integrity verification, digital signatures, and encryption domains. In our study, the utilization of the MD5 function forms an intermediary ciphertext mechanism, enhancing the encryption performance of the data. This approach not only bolsters data security but also ensures the integrity and validation during data transmission.

**[Comment 7]** In line 333, the variable i is not italicized. Therefore, it is strongly advised that the authors carefully review the entire text to avoid such oversights.

**Response:** Thank you for your careful observation. We have conducted a thorough check of the entire manuscript and rectified similar issues, including the non-italicized variable "i" in line 333.

**[Comment 8]** While considering security, encryption efficiency is also an important factor that researchers must consider. Therefore, it is recommended that the authors analyze and discuss the encryption efficiency of the proposed scheme.

**Response:** Thank you for the suggestion. We have conducted experiments and included an analysis of the encryption efficiency of the proposed scheme in our discussion.

**[Comment 9]** It is recommended that the authors adjust and improve the content of the conclusion section.

**Response:** Thank you for the suggestion. We have adjusted and improved the content of the conclusion section.

**[Comment 10]** Is there room for further refinement or improvement of the current work in the future? In other words, what is your future further work related to this manuscript?

**Response:** In the future, we plan to apply the encryption algorithm proposed in this research to practical scenarios. Our focus will be on assessing the performance, security, and scalability of the algorithm in real-world applications, seeking further enhancements and optimizations.

**[Comment 11]** The authors are advised to carefully proofread and standardize their references in accordance with the template.

**Response:** Thank you for the suggestion. We have made careful revisions and standardized the references according to the template requirements.

**Response to Reviewer 2**

**[General Comment]** The authors propose an image encryption method which uses DWT and two dynamic feedback encryptions (plaintext and intermediate ciphertext). The article describes the encryption and decryption process in detail and presents the experimental data well with images and tables.

**Response:** Thank you very much for your previous comments that helped us improve this manuscript. We have carefully studied your suggestions and have tried our best to address each of these comments. We hope the manuscript has been improved accordingly.

**[Comment 1]** Are a and b in equations (1) and (3) the same? If they are the same, why is the description of them inconsistent?

**Response:** Thank you for your inquiry. The 'a' and 'b' in equations (1) and (3) are not the same. We have updated the descriptions to ensure accuracy and consistency.

**[Comment 2]** The author's description of equation (3) is vague. For example, why are initial values assigned to a and b: 0.5 and 2 respectively? m(x) is in decimal or hexadecimal? What is the significance of such data processing?

**Response:** Thank you for your attention. The initial values of 'a' and 'b' are restricted by the parameter range of the chaotic system. 'm(x)' is generated by the MD5 function and represented in hexadecimal. The significance of this data processing lies in its association with plaintext, enhancing resistance against chosen-plaintext attacks. We have revised the description in the manuscript to better articulate these concepts.

**[Comment 3]** How efficient is the encryption and decryption?

**Response:** Thank you for your inquiry. We have conducted experiments to assess the efficiency of encryption.

**[Comment 4]** What are the advantages of two-layer dynamic feedback encryption over single-layer? How effective is the algorithm if it uses only plaintext feedback or intermediate ciphertext feedback? Please add this aspect in the experimental section.

**Response:** The two-layer dynamic feedback encryption offers advantages such as enhanced encryption performance and stronger resistance against chosen-ciphertext attacks compared to single-layer encryption. Manuscripts have been added for related experiments.

**[Comment 5]** The experimental data needs to be compared with recent articles, and it is clear that some articles are not appropriate, e.g., Ref.[49] and Ref.[50].

**Response:** Thank you for your guidance. We have revised the references to ensure the experimental data aligns with recent articles.

**[Comment 6]** Why is there no reference in the Sensitivity analysis section?

**Response:** Thank you for your reminder. In the Sensitivity Analysis section, we primarily focused on exploring the inherent characteristics of the data and did not reference other literature. The data presented in this section shows results that are very close to the ideal values.

**[Comment 7]** The original manuscript mentions four innovations, including the use of DWT, in the introduction, but not in the conclusion?

**Response:** Thank you for pointing that out. We have made modifications to the conclusion section to ensure it covers the innovations mentioned in the introduction, including the use of DWT.

**[Comment 8]** In addition, the manuscript needs to be revised in the following ways:

1. Show the full name of the 2D-SLMM chaotic system as early as possible, not in line 93.

2. Change the abbreviations of Figure and Equation. They should be Fig. and Eq..

3. In Figure 2, Seq1 should be S1, and the others should be the same.

4. Use abbreviation for Figure at line 273.

5. Unify the format of the literature.

6. The source of the pictures used in the experiment should be stated.

**Response:** Thank you for the detailed guidance. We have thoroughly checked and made the following modifications to the manuscript:

1.We have presented the full name of the 2D-SLMM chaotic system at an earlier point, not limited to line 93.

2.The abbreviations for figures and equations have been changed to Fig. and Eq..

3.In Figure 2, 'Seq1' has been updated to 'S1', and the corresponding adjustments have been made for others.

4.The abbreviation for 'Figure' has been used at line 273.

5.We have standardized the format of the references.

6.The sources of the images used in the experiments have been indicated.
