# Supplementary material for: Secure image communication based on two-layer dynamic feedback encryption and DWT information hiding
Source: PLoS One. 2024 Aug 27;19(8):e0300228. doi: 10.1371/journal.pone.0300228 (PMC11349087; doi:10.1371/journal.pone.0300228)
Supplement: Attachment — Submitted filename: Response.docx [file pone.0300228.s002.docx]

**Response to Reviewer 1**

**[Comment] With the efforts of the authors, the quality of the paper has been significantly improved. Therefore, the paper can be accepted for publication.**

**Response:** We express our heartfelt gratitude for your insightful and constructive comments during the review process. Your professional guidance is of great significance to our research work. Following your valuable suggestions, we have thoroughly revised and improved the paper. This includes a deeper exploration of data analysis, optimization of the paper's structure, and strengthening of the arguments' persuasiveness. We believe these modifications not only enhance the quality of the paper but also make it more in line with the standards of academic journals. Once again, we thank you for your precious time and professional opinions.

**Response to Reviewer 3**

**[Comment 1]** The authors used MD5 to generate hash values and employed it to create the secret key, utilizing MD5 twice-once for round 1 and again for round 2. Firstly, the secret key should be larger than what they calculated because they will share 128 bits for each round with the receiver.l disagree with this approach as it deviates from the principles of cryptography.

**Response:** Thank you for your review and valuable suggestions. Regarding your concern about using MD5 for key generation, I have carefully considered and found it quite enlightening. I realize that continuous updating and improvement are crucial in the field of cryptography. Therefore, in the future, I will dedicate myself to researching and applying more secure and innovative encryption technologies to ensure the safety and integrity of data.

**[Comment 2]** The authors should calculate PSNR because they employed frequency transformation in the encryption algorithm. The permutation step is not clear, it's essential to specify whether it's a bit permutation. lf it is, the speed of the permutation operation needs to be addressed, as it might take more time compared to pixel permutations, This aspect needs to be highlighted further.

**Response:** Thank you for your valuable comments. We have added PSNR experimental results and clearly stated that bit-level permutation is used, enhancing the completeness and accuracy of our paper.

**[Comment 3]** 2D chaotic maps require time, and l believe this is not included in the time calculation, Additionally, one measurement may not be sufficient for it. It would be beneficial to review some latest articles and expand the comparison to demonstrate the superiority of the paper.

**Response**: Thank you for your suggestions. We have supplemented the calculation of the overall encryption time of the algorithm, reviewed related literature again, and expanded comparative experiments to demonstrate the advantages of our paper.
